# Vitamin C ameliorates tetrahydrocannabinol-induced spermatotoxicity in-vitro

**DOI:** 10.1186/s40795-020-00387-y

**Published:** 2020-11-24

**Authors:** Abdullateef Isiaka Alagbonsi, Luqman Aribidesi Olayaki

**Affiliations:** 1 Department of Physiology, School of Medicine and Pharmacy, University of Rwanda College of Medicine and Health Sciences, Huye, Republic of Rwanda; 2 Department of Physiology, College of Health Sciences, University of Ilorin, Ilorin, Kwara Nigeria

**Keywords:** Kinematics, Spermatozoa motility, Spermatotoxicity, Tetrahydrocannabinol, Vitamin C

## Abstract

**Background:**

We investigated the in-vitro effects of vitamin C on delta-9-tetrahydrocannabinol (THC) -induced reduction in spermatozoa motility and kinematics.

**Methods:**

Six rats were used for the study. Semen from each of the 6 rats was randomly divided into 6 groups such that each rat’s semen was in all of the groups. Groups I-III received placebo, THC (1 mM), and vitamin C (5 mM) respectively. Group IV was pre-treated with cannabinoid receptors’ blockers (CBs^−^) 1 and 2, followed by THC. Groups V and VI received THC and vitamin C, but group VI was additionally pre-treated with CBs^−^.

**Results:**

The spermatozoa progressive motility, average path velocity (VAP), curvilinear velocity (VCL), straight-line velocity (VSL), amplitude of lateral head (ALH) and beat cross frequency (BCF) were reduced by THC (6.08 ± 1.16%; 5.64 ± 0.82 μm/s; 6.96 ± 0.74 μm/s; 2.75 ± 0.23 μm/s; 0.31 ± 0.02 μm; and 0.78 ± 0.08 Hz respectively) but increased by vitamin C (51.20 ± 1.32%; 17.90 ± 0.21 μm/s; 25.11 ± 0.96 μm/s; 8.80 ± 0.27 μm/s; 0.75 ± 0.01 μm; and 3.15 ± 0.03 Hz respectively) when compared to control (39.72 ± 0.38%; 13.70 ± 0.29 μm/s; 18.04 ± 0.58 μm/s; 7.54 ± 0.34 μm/s; 0.65 ± 0.02 μm; and 2.79 ± 0.01 Hz respectively). Vitamin C inhibited the THC-induced reduction in these parameters (37.36 ± 0.73%; 10.98 ± 0.45 μm/s; 13.58 ± 0.30 μm/s; 7.11 ± 0.22 μm/s; 0.58 ± 0.01 μm; and 2.60 ± 0.01 Hz respectively) in the absence of CBs^−^ 1 and 2, and even caused additional increases in progressive motility (49.54 ± 1.01%), VAP (15.70 ± 0.38 μm/s) and VCL (22.53 ± 0.29 μm/s) above the control levels with CBs^−^.

**Conclusion:**

Vitamin C ameliorates the THC-induced reduction in spermatozoa motility in-vitro by modulation of their kinematics.

## Background

Marijuana, preparation of the flower, leaves and seeds of the plant *Cannabis sativa* (CS), is the most widely abused illicit drug worldwide [1] and is becoming globally legalised due to its medicinal use by patients with pain [2], inflammation [3], epilepsy [4], cancer [5], etc. However, its detrimental effects on male reproductive functions have been well-established. For instance, it decreases germ cell proliferation, reproductive organ weight [6, 7], and semen parameters [7–9] by eliciting endocrine disruption, hyperprolactinaemia [10], down-regulation of the hypothalamic-pituitary-gonadal axis [10, 11], and oxidative stress [9].

Delta-9-tetrahydrocannabinol (THC, an active psychoactive compound in CS), prescribed under the name Dronabinol (synthetic THC in sesame oil), is clinically being used for the treatment of acquired immunodeficiency syndrome (AIDS) -associated cachexia, multiple sclerosis, and cancer chemotherapy-associated nausea [12, 13]. However, it has been consistently shown to negatively alter spermatozoa motility, swimming behaviour, and acrosome reaction in sea urchin [14–16], rat [17], and human [18, 19].

The effects of vitamin C on spermatozoa parameters and fertility rate in subfertile males remain controversial. For instance, its protective effect against testicular damage has been well-reported in different conditions [20, 21], while its toxic [20] and deoxyribonucleic acid (DNA)- damaging [22, 23] potentials have also been shown.

We have previously observed in our in-vivo studies that vitamin C exacerbates the CS-induced gonadotoxicity, oxidative stress and reproductive hormonal toxicities, but these CS-induced toxicities were ameliorated only when vitamin C was co-administered with melatonin [7, 9, 10]. In our very recent in-vitro study, we observed that melatonin alone ameliorated THC-induced spermatotoxicity by attenuating the THC-induced reduction of hyper-activated motility (HAM) in the capacitated spermatozoa [17]. The results of our recent in-vitro work [17] showed that melatonin alone attenuated, but did not abolish, the THC-induced reduction in spermatozoa motility and kinematics. This lack of abolishment is evident as the motility and kinematics were still significantly lower in spermatozoa that were treated with a combination of melatonin and THC than the control.

Vitamin C has been reported to increase the motility and kinematics of caprine epididymal spermatozoa in-vitro [24]. Since the combination of melatonin and vitamin C abolished the spermatotoxic effects of CS in-vivo*,* the lack of abolishment of the spermatotoxic effect of THC by melatonin alone in-vitro suggests that the effect of melatonin and vitamin C on the semen parameters might be additive or synergistic. This speculation led us to also investigate the effect of vitamin C on spermatozoa motility and kinematics in this study to clear our doubts whether vitamin C contributed to the combined effect of melatonin and vitamin C or whether the combined effect is a result of melatonin only.

Following the procedures used in our previous in-vitro study, the present study investigated the effect of vitamin C on motility and kinematics of capacitated rat spermatozoa treated with cannabinoids using the computer-assisted sperm analyser (CASA). We hypothesised that vitamin C will ameliorate the THC-induced reduction in spermatozoa motility and kinematics.

## Methods

### Experimental protocol

Six [6] male Wistar albino rats (200–210 g) sourced from the Department of Biochemistry’s Animal House, University of Ilorin, Nigeria were housed at room temperature under the daily light/dark cycle with free access to food and water ad libitum. In addition to the study approval by the Ethics Committee of the University of Ilorin, the “Principles of laboratory animal care (NIH publication No. 85-23, revised 1985)” were followed.

Following the sacrifice of the rats with 0.8 ml kg^− 1^ body weight of ketamine hydrochloride, the cauda epididymides were rapidly removed and minced in the modified Biggers–Whitten–Whittingham (BWW) capacitation medium, whose components have been previously described [17, 25]. The liberated spermatozoa were incubated in the medium. Dimethyl sulphoxide (DMSO) was used to prepare the stock solution of the drugs, which was then diluted to the required concentration before each experiment in a BWW capacitation medium such that the final DMSO concentration was 0.2% (Vol/Vol) [25] that does not affect spermatozoa motility [17].

Following the method of Charan and Kantharia [26] for the determination of animal sample size, semen from each rat (*n* = 6) was randomly divided into 6 treatment groups such that each rat’s semen was in all of the groups as follows: Groups I-III were respectively treated with placebo (control mixture), THC (1 mM), and vitamin C (5 mM). Group IV was pretreated with 1 mM each of CBs^−^ 1 (SR141716) and 2 (AM-630), followed by THC (1 mM). Groups V and VI were both treated with THC and vitamin C, but group VI was additionally pretreated with 1 mM each of CBs^−^ 1 (SR141716) and 2 (AM-630). Experiments were carried out by incubating spermatozoa in the BWW capacitation medium for 30 min. We generated dose-response and time-course of modulation curves from our preliminary experiment to arrive at the doses of THC and vitamin C and the duration used for the study while the same dose of THC was used for the CBs^−^ [17].

### Determination of spermatozoa motility and kinematics

Spermatozoa progressive motility and kinematics (average-path velocity [VAP], curvilinear velocity [VCL], straight-line velocity [VSL], the amplitude of lateral head displacement [ALH], beat cross frequency [BCF], wobble, straightness, and linearity) were recorded with CASA (JH-6004 Sperm Quality Analyser, Jiangsu Jiahua Electronic Instrument Co., Ltd., Jiangsu, China) as previously described [17].

### Data analysis

Data were blindly analysed using statistical software for the social sciences (SPSS) version 16.0 for Windows (IBM Corporation, Armonk, NY, USA). All values given were the Mean ± S.E.M of the variables. The normality of the data was assessed using the Shapiro-Wilk test. Significance was assessed by the one-way analysis of variance (ANOVA), followed by a posthoc Tukey test for multiple comparisons. The five experimental groups were compared with the control group that received placebo, while groups that received cannabinoids with (out) vitamin C were additionally compared with THC group. Receiver Operating Characteristic (ROC) curve was used to examine the predictive abilities of spermatozoa kinematics on their progressive motility. The *p*-values of 0.05 or less were considered statistically significant.

## Results

### Dose-response and time-course of modulation of spermatozoa motility by tetrahydrocannabinol and vitamin C

**Fig. 1:**
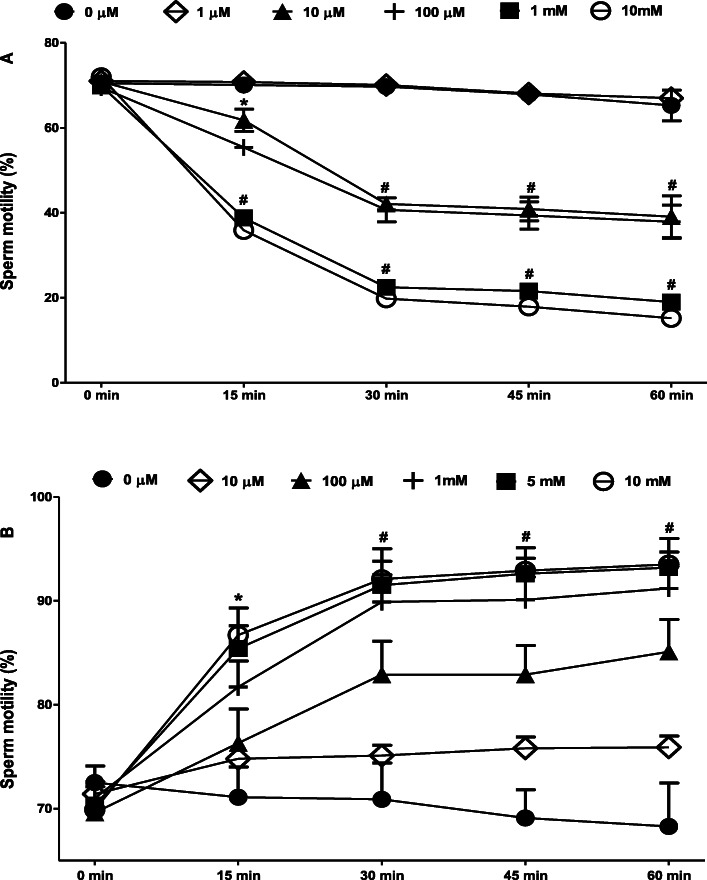
Dose-response and time-course of modulation of the percentage motility (progressive + non-progressive) of spermatozoa after incubation in tetrahydrocannabinol (**a**) or vitamin C (**b**). ^*^p < 0.01; ^#^*p* < 0.001 vs. Baseline (0 min)

Throughout the observation period, 10 μM, 100 μM, 1 mM and 10 mM but not 1 μM of THC reduced the rat spermatozoa motility when compared to the baseline (Fig. 1a). On the contrary, 100 μM, 1 mM, 5 mM, and 10 mM but not 10 μM of vitamin C increased the rat spermatozoa motility when compared to the baseline (Fig. 1b).

### THC-induced reduction in progressive motility is attenuated by vitamin C

**Fig. 2:**
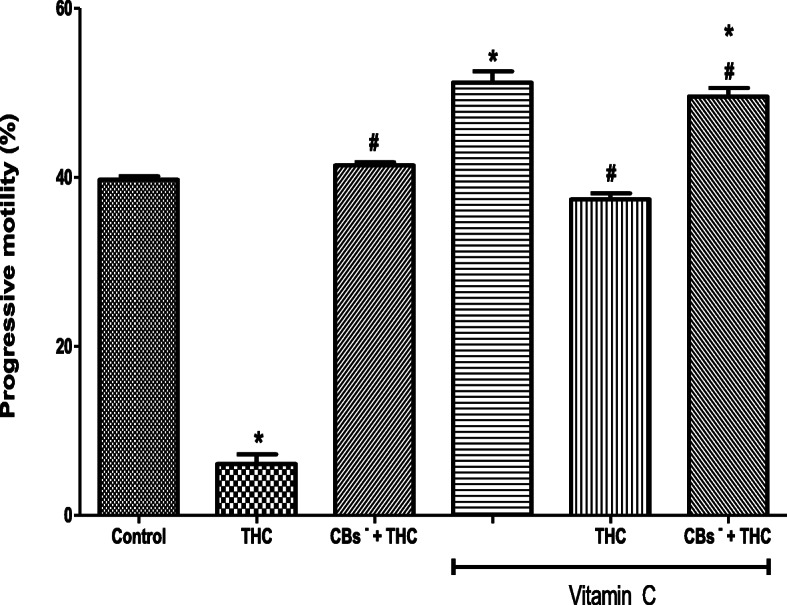
Vitamin C abolished the tetrahydrocannabinol-induced reduction in the progressive motility of rat spermatozoa. THC, tetrahydrocannabinol; CBs^−^, cannabinoid receptors’ blockers; ^*^p < 0.05 vs. Control; ^#^*p* < 0.05 vs. THC

THC reduced the spermatozoa progressive motility (6.08 ± 1.16%) when compared to the control (39.72 ± 0.38%) but this reduction was inhibited by cannabinoid receptors 1 and 2 blockers (41.38 ± 0.36%). The progressive motility was increased by vitamin C (51.20 ± 1.32%) when compared to the control (39.72 ± 0.38%). Interestingly, vitamin C inhibited the THC-induced reduction in progressive motility (37.76 ± 0.73%), maintaining it at 94% of the control value. When cannabinoid receptors 1 and 2 were blocked, vitamin C completely abolished the effect of THC on the progressive motility and even caused its additional increase (49.54 ± 1.01%) by 20% above the control level (Fig. 2).

### THC-induced reductions in some spermatozoa kinematics are attenuated by vitamin C

**Fig. 3:**
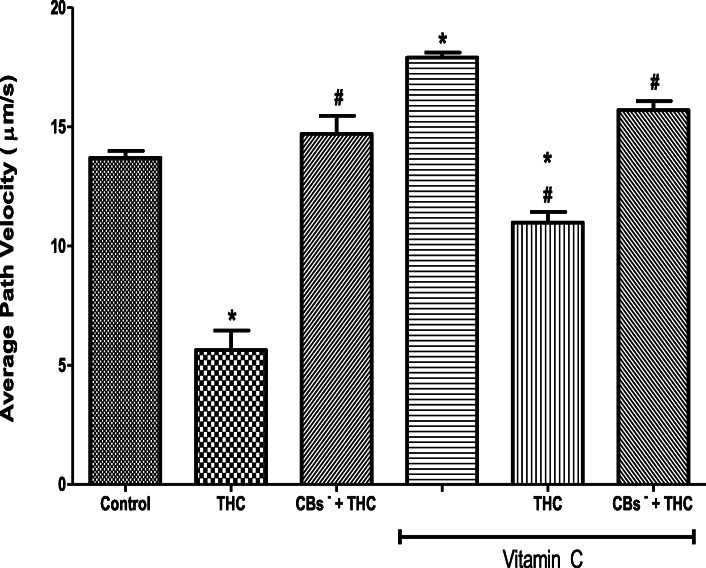
Vitamin C attenuated the tetrahydrocannabinol-induced reduction in the average path velocity of rat spermatozoa. THC, tetrahydrocannabinol; CBs^−^, cannabinoid receptors’ blockers; ^*^p < 0.05 vs. Control; ^#^*p* < 0.05 vs. THC

THC reduced the spermatozoa VAP (5.64 ± 0.82 μm/s) when compared to the control (13.70 ± 0.29 μm/s) but this reduction was inhibited by cannabinoid receptors 1 and 2 blockers (14.70 ± 0.76 μm/s). The VAP was increased by vitamin C (17.90 ± 0.21 μm/s) when compared to the control (13.70 ± 0.29 μm/s). Moreover, vitamin C inhibited the THC-induced reduction in VAP (10.98 ± 0.45 μm/s), maintaining it at 80% of the control. When cannabinoid receptors 1 and 2 were blocked, vitamin C completely abolished the effect of THC on VAP and even caused its additional increase (15.7 ± 0.38 μm/s) by 13% above the control level (Fig. 3).

**Fig. 4:**
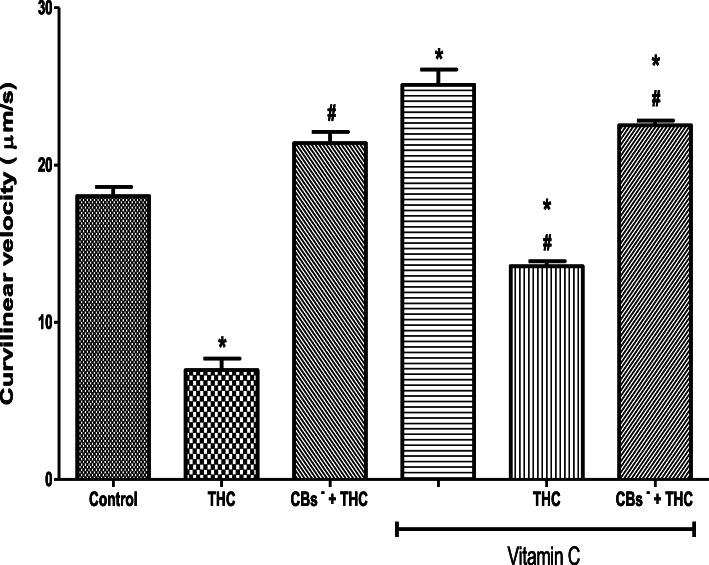
Vitamin C attenuated the tetrahydrocannabinol-induced reduction in the curvilinear velocity of rat spermatozoa. THC, tetrahydrocannabinol; CBs^−^, cannabinoid receptors’ blockers; ^*^p < 0.05 vs. Control; ^#^*p* < 0.05 vs. THC

THC reduced the spermatozoa VCL (6.96 ± 0.74 μm/s) when compared to the control (18.04 ± 0.58 μm/s) but this reduction was inhibited by cannabinoid receptors 1 and 2 blockers (21.39 ± 0.73 μm/s). The VCL was increased by vitamin C (25.11 ± 0.96 μm/s) when compared to the control (18.04 ± 0.58 μm/s). Furthermore, vitamin C inhibited the THC-induced reduction in VCL (13.58 ± 0.30 μm/s) by maintaining it at 75% of the control value. When cannabinoid receptors 1 and 2 were blocked, vitamin C completely abolished the effect of THC on VCL and even caused its additional increase (22.53 ± 0.29 μm/s) by 20% above the control level (Fig. 4).

**Fig. 5:**
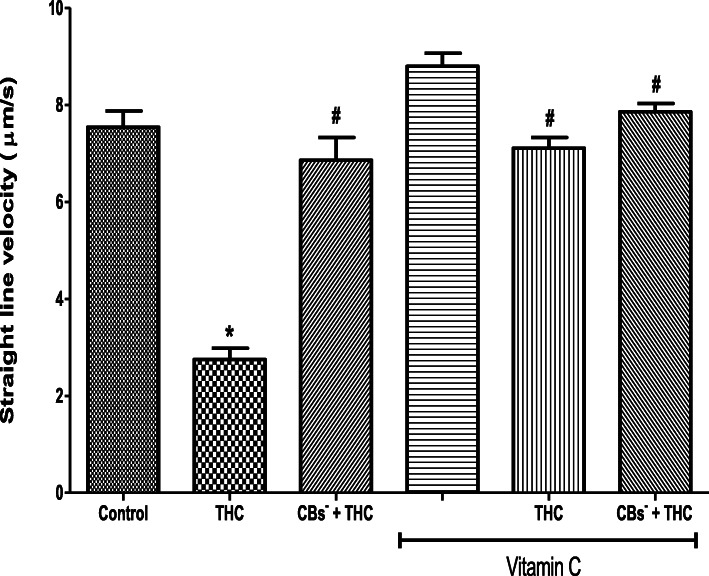
Vitamin C abolished the tetrahydrocannabinol-induced reduction in the straight line velocity of rat spermatozoa. THC, tetrahydrocannabinol; CBs^−^, cannabinoid receptors’ blockers; ^*^p < 0.05 vs. Control; ^#^*p* < 0.05 vs. THC

THC reduced the spermatozoa VSL (2.75 ± 0.23 μm/s) when compared to the control (7.54 ± 0.34 μm/s) but this reduction was inhibited by cannabinoid receptors 1 and 2 blockers (6.86 ± 0.47 μm/s). The VSL was increased, albeit insignificant, by vitamin C (8.80 ± 0.27 μm/s) when compared to the control (7.54 ± 0.34 μm/s). Vitamin C inhibited the THC-induced reduction in VSL (7.11 ± 0.22 μm/s) by maintaining it at 94% of the control value. When cannabinoid receptors 1 and 2 were blocked, vitamin C completely abolished the effect of THC on VSL by maintaining it at a level (7.86 ± 0.17 μm/s) comparable to that of the control (Fig. 5).

**Fig. 6:**
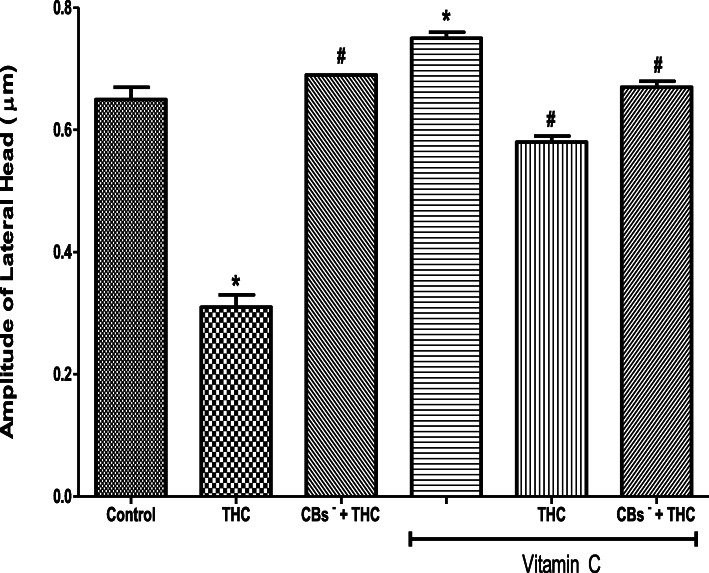
Vitamin C attenuated the tetrahydrocannabinol-induced reduction in the amplitude of lateral head of rat spermatozoa. THC, tetrahydrocannabinol; CBs^−^, cannabinoid receptors’ blockers; ^*^p < 0.05 vs. Control; ^#^*p* < 0.05 vs. THC

THC reduced the spermatozoa ALH (0.31 ± 0.02 μm) when compared to the control (0.65 ± 0.02 μm) but this reduction was inhibited by cannabinoid receptors 1 and 2 blockers (0.69 ± 0.00 μm). The VSL was increased by vitamin C (0.75 ± 0.01 μm) when compared to the control (0.65 ± 0.02 μm). Vitamin C inhibited the THC-induced reduction in ALH (0.58 ± 0.01 μm) by maintaining it at 89% of the control value. When cannabinoid receptors 1 and 2 were blocked, vitamin C completely abolished the effect of THC on ALH by maintaining it at a level (0.67 ± 0.01 μm) comparable to that of the control (Fig. 6).

**Fig. 7:**
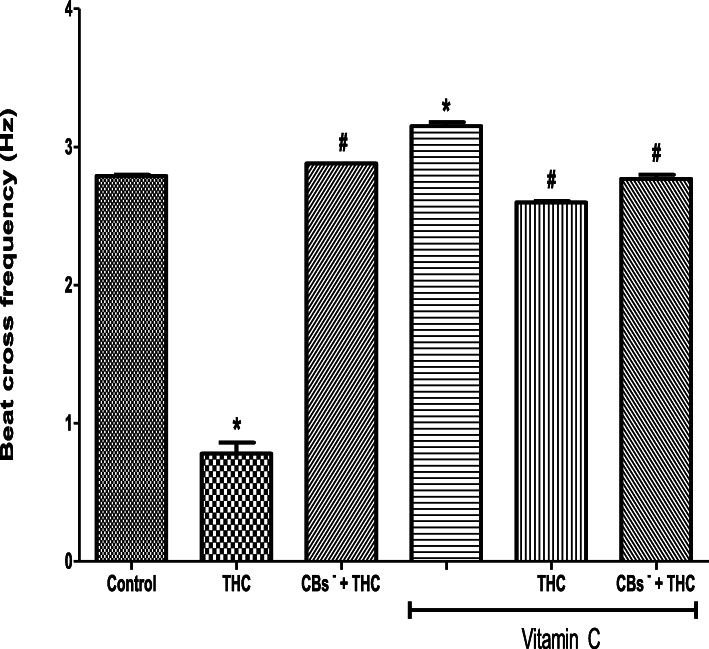
Vitamin C attenuated the tetrahydrocannabinol-induced reduction in the beat cross frequency of rat spermatozoa. THC, tetrahydrocannabinol; CBs^−^, cannabinoid receptors’ blockers; ^*^p < 0.05 vs. Control; ^#^*p* < 0.05 vs. THC

THC reduced the spermatozoa BCF (0.78 ± 0.08 Hz) when compared to the control (2.79 ± 0.01 Hz) but this reduction was inhibited by cannabinoid receptors 1 and 2 blockers (2.88 ± 0.00 Hz). The BCF was increased by vitamin C (3.15 ± 0.03 Hz) when compared to the control (2.79 ± 0.01 Hz). Vitamin C inhibited the THC-induced reduction in BCF (2.60 ± 0.01 Hz) by maintaining it at 93% of the control value. When cannabinoid receptors 1 and 2 were blocked, vitamin C completely abolished the effect of THC on BCF by maintaining it at a level (2.77 ± 0.03 Hz) comparable to that of control (Fig. 7).

### The effect of vitamin C on velocity ratios in THC-treated spermatozoa

THC and/or vitamin C caused no significant change in the spermatozoa wobble, straightness and linearity when compared to control. THC did not also have any effect on these velocity ratios when cannabinoid receptors 1 and 2 were blocked, either in the presence or absence of vitamin C (Table 1).

**Table 1:** Effect of vitamin C on velocity ratios in tetrahydrocannabinol-treated spermatozoa

Groups	Wobble (VAP/VCL, %)	Straightness (VSL/VAP, %)	Linearity (VSL/VCL, %)
Control	76.08 ± 2.91	55.18 ± 3.27	42.01 ± 3.13
THC	81.45 ± 9.23	49.81 ± 4.49	40.52 ± 5.70
CBs^−^ + THC	68.64 ± 1.43	46.89 ± 3.74	32.16 ± 2.55
Vitamin C	71.44 ± 1.98	49.14 ± 0.91	35.07 ± 0.39
Vitamin C + THC	80.96 ± 4.01	64.83 ± 1.96 ^#^	52.35 ± 1.36 ^#^
CBs^−^ + THC + Vitamin C	69.74 ± 2.59	50.15 ± 2.04	34.88 ± 0.63

*THC* tetrahydrocannabinol, *CBs*^−^ cannabinoid receptors’ blockers, *VSL* straight line velocity, *VCL* curvilinear velocity, *VAP* average-path velocity, ^#^*p* < 0.05 vs. THC

### Prediction of progressive motility by the kinematics of spermatozoa

Using the ROC curve, we determined if kinematics could predict the progressive motility of spermatozoa treated with THC and/or vitamin C. The curve showed that 100% of progressively motile spermatozoa had 8.76 μm s^− 1^ as VAP, 10.36 μm s^− 1^ as VCL, 4.96 μm s^− 1^ as VSL, 0.45 μm as ALH, 1.73 Hz as BCF, 66.49% as wobble, and 32.46% as linearity while 100% of non-progressively motile spermatozoa had below these parameters’ cut-off values (except for wobble and linearity that the specificity is 33.30%). The velocity ratios (wobble, straightness, and linearity) had lower confidence intervals and areas under the ROC curves than velocities, ALH, and BCF. Thus, there was a very low probability that the progressively motile and non-motile spermatozoa differ in their velocity ratios (wobble, straightness and linearity), while there was a very high probability that they differ in their velocities (VAP, VCL, and VSL), ALH and BCF (Table 2).

**Table 2:** Prediction of hyper-activated motility by the kinematics of capacitated spermatozoa treated with tetrahydrocannabinol with (out) vitamin C

Outcomes	Predictors	Sensitivity (%)	Specificity (%)	Cutoff value	AUC	95% CI	*p*-values
Progressive motility	VAP (μm/s)	100.00	100.00	8.76	1.000	1.000 to 1.000	0.000
	VCL (μm/s)	100.00	100.00	10.36	1.000	1.000 to 1.000	0.000
	VSL (μm/s)	100.00	100.00	4.96	1.000	1.000 to 1.000	0.000
	ALH (μm)	100.00	100.00	0.45	1.000	1.000 to 1.000	0.000
	BCF (Hz)	100.00	100.00	1.73	1.000	1.000 to 1.000	0.000
	Wobble (%)	100.00	33.30	66.49	0.333	−0.200 to 0.867	0.405
	Straightness (%)	88.90	66.70	48.43	0.778	0.455 to 1.101	0.166
	Linearity (%)	100.00	33.30	32.46	0.593	0.181 to 1.004	0.644

*VAP* Average-path velocity, *VCL* Curvilinear velocity, *VSL* Straight-line velocity, *ALH* Amplitude of lateral head displacement, *BCF* Beat/cross frequency, *AUC* Area under the receiver operating characteristic curve, *CI* Confidence interval

## Discussion

The reduction in spermatozoa motility by THC was initially reported in sea urchin by Schuel’s group [14–16]. A series of studies later showed that cannabinoid receptors activation by anandamide (an endocannabinoid agonist) or THC inhibits spermatozoa functions like motility, velocities, capacitation and acrosome reaction in the frog, rat [6, 17], boar [27], and human [19, 28–31]. Furthermore, the spermatotoxic potential and the consequent anti-fertility effect of marijuana [7, 9, 10] and its primary psychoactive cannabinoid (THC) [17] are well-known.

Ejaculated spermatozoa capacitation process in the female reproductive tract involves membrane and metabolic modifications such as the generation of reactive oxygen species (ROS), an increase in intracellular ions and protein tyrosine phosphorylation, and changes in motility and plasma membrane fluidity [32, 33]. Events like spermatozoa binding to the zona pellucida (ZP), acrosome reaction, and oocyte fertilisation all depend on successful capacitation process [34], prevention of which will lead to fertilisation failure [35]. During this process, spermatozoa acquire a motility pattern called HAM [36] that is characterised by asymmetrical flagellar beating [37], which is needed for fast swimming and generation of enough force necessary to penetrate cumulus cells and ZP during fertilisation [38]. Because the HAM patterns of mammalian spermatozoa capacitated in-vivo and in-vitro have been reported to be similar [39, 40], it has been very easy for researchers to study different aspects of spermatozoa physiology by in-vitro capacitation. Our data in this study showed that THC reduced the progressive motility of spermatozoa incubated in capacitation medium, which corroborates its spermatotoxic effect.

Vitamin C has been previously reported to improve rat’s semen parameters in-vivo [9] and ameliorate the spermatotoxicity induced by cisplatin in rats [41, 42] and p-dimethylaminoazobenzene in mice [43]. In a randomised controlled clinical trial that included cases after varicocele surgery, vitamin C supplementation significantly increased motility and morphology but not count in infertile young adult males with low-quality spermatozoa, suggesting that it positively affects qualitative but not quantitative characteristics of semen analysis in this condition [44]. Apart from the reported improvement in spermatozoa quality in smokers by increasing vitamin C dose from 200 to 1000 mg [45], another observational study on healthy, non-smoking Americans showed that vitamin C-containing diet or supplement increased spermatozoa count and motility [46]. When combined with vitamin E, vitamin C has also been reported to enhance rabbit male fertility [47] by increasing concentration and total motile but decreasing abnormal and dead spermatozoa [47, 48]. A combination of vitamins C and E reportedly ameliorate the oxidative stress and spermatotoxicity induced by endosulfan in rats [49].

Does vitamin C have spermatoprotective effect in-vitro? Previous reports have shown that vitamin C is the most important seminal anti-oxidant, accounting for about 65% of the seminal anti-oxidant capacity, and is currently being used in-vitro to enhance spermatozoa quality in infertility clinics [50, 51]. An Egyptian study has linked smoking to decrease in seminal plasma vitamin C, which was shown to consequently reduce semen parameters and fertilisation potential [52]. Our dose-response and time-course of modulation study showed that vitamin C increased the percentage motility of spermatozoa in a dose-dependent but not time-dependent manner. For instance, 100 μM, 1 mM, 5 mM, and 10 mM, but not 10 μM, significantly increased the spermatozoa motility throughout the observation period when compared to the baseline. Furthermore, incubation of spermatozoa in 5 mM of vitamin C solution also increased their motility by 22% when compared to control. The vitamin C-induced increase in spermatozoa motility was associated with an increase in VAP, VCL, VSL, ALH and BCF but decrease in straightness and linearity. These data provide pieces of evidence that vitamin C increases motility by enhancing the kinematics of spermatozoa. These data are consistent with the previous report of Babaie and Sivandi [24] that also reported an improvement in the motility and kinematics of caprine spermatozoa treated with vitamin C in-vitro. Similar to our study, Babaie and Sivandi [24] obtained epididymal spermatozoa from adult goat testes and incubated them in the capacitation medium (Ca^2+^-free Tyrode’s medium, spermatozoa TALP) supplemented with 50 μg/ml vitamin C, after which the motion parameters were assessed using the CASA at various intervals. They also observed that vitamin C increased total and progressive spermatozoa motility, VCL, VSL, linearity, ALH and BCF.

Can vitamin C ameliorate the THC-induced spermatotoxicity? In our previous in-vivo studies, we reported that the CS-induced spermatotoxicities (and the associated endocrine disruption and oxidative stress) were exacerbated by either vitamin C or melatonin when administered separately, but ameliorated when vitamin C was co-administered with melatonin [7, 9, 10]. However, our most recent in-vitro study showed that melatonin attenuated THC-induced reduction in spermatozoa motility and kinematics of rat. Based on our observation that the modulatory effects of melatonin on spermatotoxicity induced by CS or THC are different in-vivo and in-vitro, we were interested to know the effect of vitamin C on THC-induced spermatotoxicity in-vitro. In the present in-vitro study where spermatozoa were incubated with THC and vitamin C in the capacitation medium, we observed for the first time that vitamin C completely abolished the THC-induced reduction in progressive motility of spermatozoa by maintaining it at 94% of the control (which is not significantly different from the control level). We also observed that vitamin C attenuated the THC-induced reduction of VAP, VCL, ALH, and BCF but abolished the THC-induced reduction in VSL. These data provide pieces of evidence that vitamin C ameliorates the THC-induced spermatotoxicity, especially as regards motility and kinematics of spermatozoa. Taken together with our previous in-vivo study, it also suggests that vitamin C ameliorates cannabinoid-induced spermatotoxicity in-vitro but exacerbates it in-vivo*.* It is thus similar to our recent study where we reported that melatonin ameliorates cannabinoid-induced spermatotoxicity in-vitro but exacerbates it in-vivo [17].

Is there a crosstalk between vitamin C and cannabinoid signalling? The existence of cannabinoid receptors (CbRs) and the endocannabinoid system in mammalian spermatozoa has been well-reported [31, 53, 54]. We have reported that THC-induced reduction in the HAM of spermatozoa was abolished when both CbRs 1 and 2 were blocked, but was only attenuated when either CbR 1 or CbR 2 was selectively blocked. We also showed that CbR 1 contributed more than CbR 2 in THC-induced spermatotoxicity [17]. Since the involvement of both CbRs in the regulation of spermatozoa motility has been established, the current study further investigated if the lack of complete abolishment of some kinematics by vitamin C is associated with CbRs activation by THC in spermatozoa incubated with a combination of THC and vitamin C. We blocked both CbRs (SR141716 as CB^−^ 1 and AM-630 as CB^−^ 2) and then added THC and vitamin C to the capacitation medium. We observed that prior blockade of CbRs cancelled the spermatotoxic effect of THC as vitamin C abolished the effect of THC on VSL, ALH and BCF, and even raised the progressive motility, VAP and VCL above the control value. This shows that vitamin C caused more inhibition of THC-induced spermatotoxicity when CbRs were blocked and is a pointer to the fact that there is a crosstalk between vitamin C and cannabinoid signalling.

Is the ameliorative effect of vitamin C on motility related to the kinematics of spermatozoa? Across a wide range of animals like birds [55], fishes [56] and mammals [57], the competition of spermatozoa to fertilise the ovum is favoured by traits (e.g. kinematics) that promote the fertilising ability of male. To penetrate different barriers along its track, the spermatozoon requires a high flagellar BCF [58]. The VCL and ALH signify the hyper-activated motility (HAM) of spermatozoa and the high-energy state required for successful fertilisation [59] while motility and velocities of spermatozoa reflect their mitochondrial function and energy status that have been correlated with their fertility [60]. Moreover, the progressive motility of spermatozoa has been positively correlated with the VAP, VCL, VSL, ALH, BCF, straightness, and linearity but negatively correlated with wobble in bulls [57, 61, 62]. In our recent study, velocities (VAP, VCL and VSL), ALH and BCF showed strong predictive power on HAM, suggesting that these kinematics determine the response of spermatozoa capacitation-induced HAM to cannabinoids and melatonin [17]. In the present study, there was a very low probability that the progressively motile and non-motile spermatozoa differ in their velocity ratios (wobble, straightness and linearity), while there was a very high probability that they differ in their velocities (VAP, VCL, and VSL), ALH and BCF. These suggest that velocities, ALH and BCF determine the modulation of spermatozoa capacitation-induced HAM by cannabinoids and vitamin C.

Even though hydrogen peroxide is the major ROS produced in spermatozoa, a previous study has shown that 87% of infertile patients have superoxide anion [63]. Spermatozoa are sensitive to assault from free radicals due to the existence of high membrane polyunsaturated fatty acids and lack of capacity for DNA repair [64]. Increased ROS level has also been shown to correlate with decreased motility [65] and many structural and functional abnormalities of spermatozoa. Meanwhile, non-enzymatic antioxidants like vitamin C, pyruvate, vitamin E, taurine, vitamin A, hypotaurine, albumin, ubiquitol, urate, and enzymatic antioxidants like catalase, superoxide dismutase, and glutathione peroxidase/reductase have been reported to be contained in the seminal fluid [66], even though vitamin C is considered as the major antioxidant in the testis [67]. Furthermore, the ROS-induced damage in spermatozoa has been shown to reduce with supplementation of the latter’s clinical media with antioxidant [64]. Despite the physiological significance of vitamin C in the body, especially on spermatozoa, humans (unlike most animals) are unable to synthesise vitamin C, making its inclusion in diet or supplement a necessity [68]. Vitamin C transporters have been reported to be expressed in the Sertoli cells, spermatocytes, spermatids, spermatozoa and the testis [69, 70]. However, our present study is limited due to our inability to establish the relationship between vitamin C transporters and cannabinoid receptors as regards HAM of capacitated spermatozoa, which is worthy of consideration in a future study. Another limitation of our study is our inability to estimate the seminal ROS and antioxidant levels in all the groups, which would have enabled us to understand the relationship between spermatozoa kinematics and redox system and their modulation by vitamin C and/or cannabinoid system. Notwithstanding the limitations, our study is the first to provide information on the in-vitro effect of vitamin C on hyperactivated motility and kinematics of capacitated spermatozoa treated with cannabinoids.

## Conclusion

In conclusion, our present acute study shows that vitamin C ameliorates THC-induced reduction in spermatozoa motility in-vitro by modulation of their kinematics. Since the success rate of in-vitro fertilisation among users of CS is known to be low [71], the outcome of our present acute study is of clinical importance as it suggests that the success rate of in-vitro fertilisation among CS users could be improved by in-vitro supplementation of their spermatozoa with vitamin C. We recommend that a similar study should be conducted on human spermatozoa.

## Data Availability

The datasets used and/or analysed during the current study are available from the corresponding author on reasonable request.

## Abbreviations

THC: tetrahydrocannabinol
CBs^−^: cannabinoid receptors’ blockers
VAP: average path velocity
VCL: curvilinear velocity
VSL: straight-line velocity
ALH: the amplitude of lateral head
BCF: beat cross frequency
CS: *Cannabis sativa*
AIDS: acquired immunodeficiency syndrome
DNA: deoxyribonucleic acid
HAM: hyper-activated motility
CASA: computer-assisted sperm analyser
BWW: Biggers–Whitten–Whittingham
BSA: bovine serum albumin
DMSO: dimethyl sulphoxide
ANOVA: one-way analysis of variance
ROC: Receiver Operating Characteristic
ROS: reactive oxygen species
ZP: zona pellucida
CbRs: cannabinoid receptors
